# Crystal structures of *trans*-acetyl­dicarbon­yl(η^5^-cyclo­penta­dien­yl)(1,3,5-tri­aza-7-phosphaadamantane)molybdenum(II) and *trans*-acetyl­di­carbon­yl(η^5^-cyclo­penta­dien­yl)(3,7-diacetyl-1,3,7-tri­aza-5-phosphabi­cyclo­[3.3.1]nona­ne)molyb­den­um(II)

**DOI:** 10.1107/S2056989020003679

**Published:** 2020-03-17

**Authors:** Mitchell R. Anstey, John L. Bost, Anna S. Grumman, Nicholas D. Kennedy, Matthew T. Whited

**Affiliations:** a Department of Chemistry, Davidson College, 405 N Main St, Davidson, North Carolina 28035, USA; b Department of Chemistry, Carleton College, 1 N College St, Northfield, MN 55057, USA

**Keywords:** crystal structure, phosphine, acet­yl, piano-stool complex

## Abstract

The title compounds, [Mo(C_5_H_5_)(COCH_3_)(C_6_H_12_N_3_P)(CO)_2_], (1), and [Mo(C_5_H_5_)(COCH_3_)(C_9_H_16_N_3_O_2_P)(C_6_H_5_)_2_))(CO)_2_], (2), have been prepared by phosphine-induced migratory insertion from [Mo(C_5_H_5_)(CO)_3_(CH_3_)]. The mol­ecular structures of these complexes are quite similar, exhibiting a four-legged piano-stool geometry with *trans*-disposed carbonyl ligands. The extended structures of complexes (1) and (2) differ substanti­ally. For complex (1), the molybdenum acetyl unit plays a dominant role in the organization of the extended structure, joining the mol­ecules into centrosymmetrical dimers through C—H⋯O inter­actions with a cyclo­penta­dienyl ligand of a neighboring mol­ecule, and these dimers are linked into layers parallel to (100) by C—H⋯O inter­actions between the molybdenum acetyl and the cyclo­penta­dienyl ligand of another neighbor. The extended structure of (2) is dominated by C—H⋯O inter­actions involving the carbonyl groups of the acetamide groups of the DAPTA ligand, which join the mol­ecules into centrosymmetrical dimers and link them into chains along [010]. Additional C—H⋯O inter­actions between the molybdenum acetyl oxygen atom and an acetamide methyl group join the chains into layers parallel to (101).

## Abstract 2

Solid-state structures of the title compounds are presented to show the relative effects of 1,3,5-tri­aza-7-phosphaadamantane (PTA) and 3,7-diacetyl-1,3,7-tri­aza-5-phosphabi­cyclo­[3.3.1]nonane (DAPTA) ligands on the mol­ecular and extended structure.

## Chemical context   

Cyclo­penta­dienylmolybdenum polycarbonyl complexes [Mo(C_5_H_5_)(CO)_*n*_] exhibiting ‘four-legged piano-stool’ geom­etries have been studied extensively for their electronic structure and organometallic reactivity (Kubacek *et al.*, 1982 ▸). Specifically, alkyl complexes of the form Mo(C_5_H_5_)(CO)_3_(*R*) have been shown to undergo carbonyl migratory insertion, affording acyl complexes upon exposure to L-type ligands, especially phosphines (Barnett & Treichel, 1967 ▸; Butler *et al.*, 1967 ▸). The steric bulk of the phosphine ligand strongly influences the stability of the resulting complexes, with bulkier groups exhibiting enhanced deinsertion rates (Barnett, 1969 ▸; Barnett & Pollmann, 1974 ▸).

We have previously described the solid-state structures of a number of four-legged piano-stool molybdenum acetyl complexes of the type Mo(P*R*_3_)(C_5_H_5_)(CO)_2_(COCH_3_) derived from reaction of the molybdenum methyl precursor with various phosphines (Whited & Hofmeister, 2014 ▸). We have shown that the steric bulk of the phosphine substituents impacts the mol­ecular structure of the insertion product in predictable ways, primarily evidenced through the Mo—P bond lengths and P—Mo—C bond angles (Whited *et al.*, 2012 ▸, 2014 ▸), consistent with earlier findings on reactivity. We have also shown that the use of tri(2-fur­yl)phosphine, which features heteroatoms as potential hydrogen-bond acceptors, leads to an unusual structure where the acetyl is oriented away from the Cp ring rather than toward it as in other cases (Whited *et al.*, 2013 ▸).
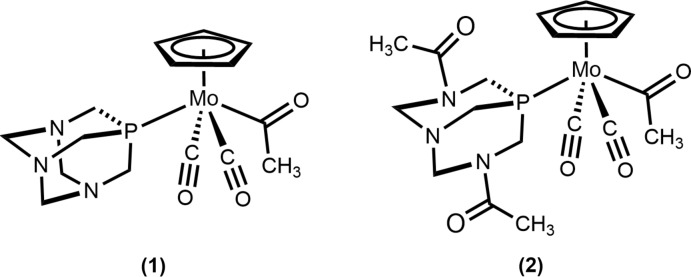


Here we report the structures of piano-stool molybdenum acetyl complexes with 1,3,5-tri­aza-7-phosphaadamantane (PTA) and 3,7-diacetyl-1,3,7-tri­aza-5-phosphabi­cyclo­[3.3.1]nonane (DAPTA) ligands featuring potential hydrogen-bond-accepting amines and carbonyl groups. The di­acetyl­ated ligand (DAPTA) shows little difference from PTA in local structure, but the introduction of acetamide groups dramatically impacts the supra­molecular organization.

## Structural commentary   

**Figure 1:**
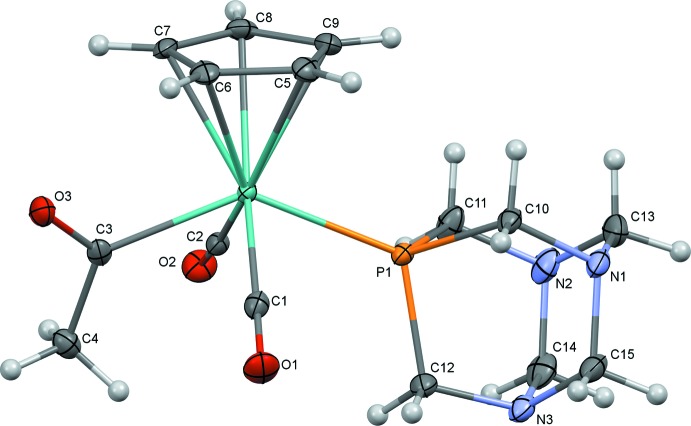
Mol­ecular structure of (1) with ellipsoids at 50% probability.

The mol­ecular structures of (1) and (2) are illustrated in Figs. 1 ▸ and 2 ▸. Both (1) and (2) exhibit a *trans* disposition of the carbonyl ligands, as seen for other compounds of this type. It was envisioned that incorporation of hydrogen-bond acceptors (amines and amides) might allow access to an alternate orientation of the acetyl group, as observed for a related structure featuring a tri(2-fur­yl)phosphine ligand, but instead the oxygen of the acetyl group points toward the Cp ring, consistent with other structures of related complexes.

Selected geometric parameters for (1) and (2) are presented in Tables 1 ▸ and 2 ▸. The Mo1—P1 bond lengths for PTA derivative (1) [2.4321 (5) Å] and DAPTA derivative (2) [2.4258 (6) Å] are similar to one another and slightly shorter than for the related complexes we have reported. This finding is consistent with the lower steric profile of the polycyclic PTA and DAPTA ligands relative to, for example, PPh_3_, PMePh_2_, and PMe_2_Ph. The most significant mol­ecular difference between (1) and (2) is seen in the C3—Mo1—P1 bond angle, which is larger for (1) [136.77 (4)°] than for (2) [133.99 (6)°].

## Supra­molecular features   

**Figure 3:**
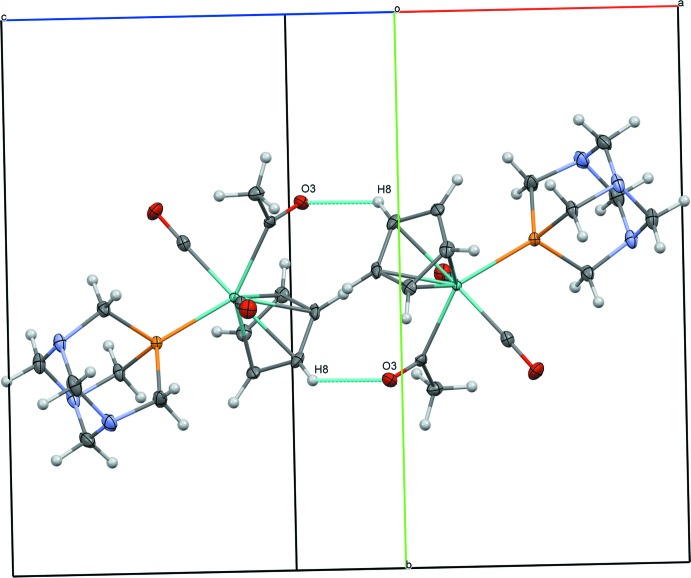
Centrosymmetrical dimer of (1), viewed perpendicular to (101).

**Figure 4:**
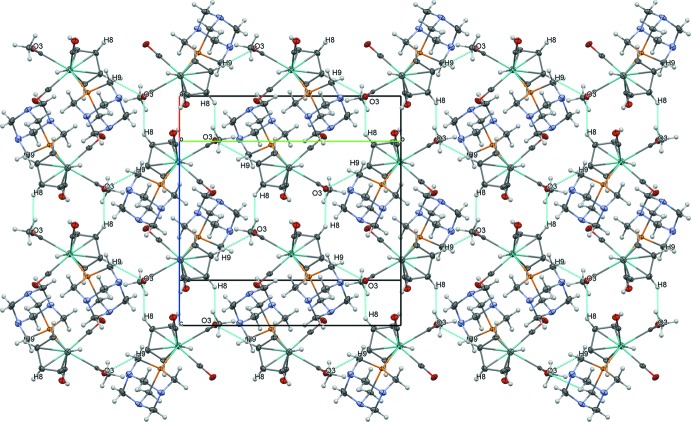
Layers of (1) formed by C—H⋯O inter­actions, viewed perpendicular to (100).

Although the mol­ecular structures of (1) and (2) are quite similar, the di­acetyl­ation of the phosphine ligand (transforming PTA to DAPTA) leads to significant differences in the extended structure. As was the case for several previously reported complexes of this sort with different phosphine ligands, the extended structure of (1) is dominated by inter­actions of atom O3 from the molybdenum acetyl. Short, non-classical C—H⋯O inter­actions between O3 and H8 of a cyclo­penta­dienyl (Cp) ring (2.49 Å) link (1) into centrosymmetrical dimers (Fig. 3 ▸). These dimers are further connected into layers parallel to (100) by non-classical C—H⋯O hydrogen bonds between O3 and H9 (2.37 Å) (Table 3 ▸, Fig. 4 ▸). Although the PTA ligand features three nitro­gen atoms as potential hydrogen-bond acceptors, these are not observed to play an important organizing structural role.

**Figure 5:**
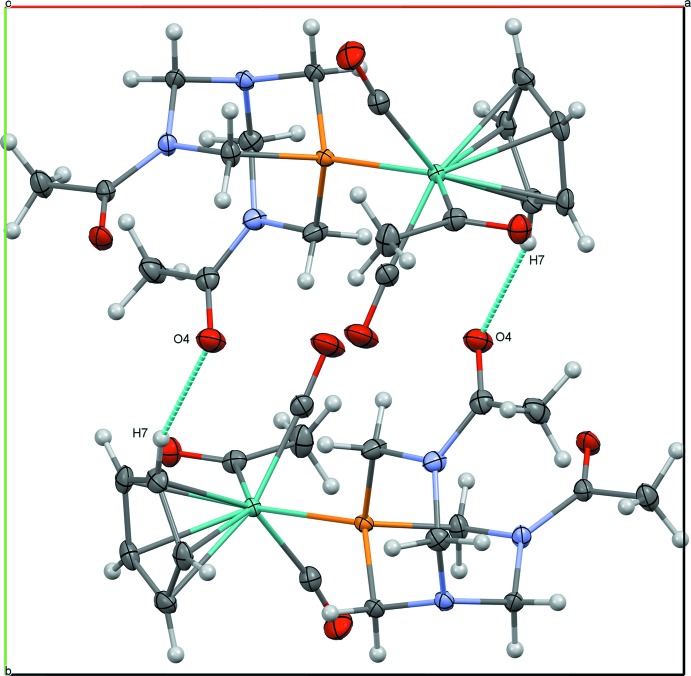
Centrosymmetrical dimer of (2), viewed along to [001].

**Figure 6:**
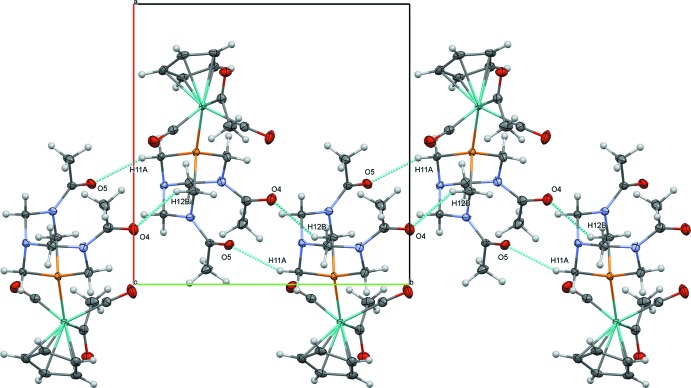
Chains of (2) along [010], viewed perpendicular to (010).

**Figure 7:**
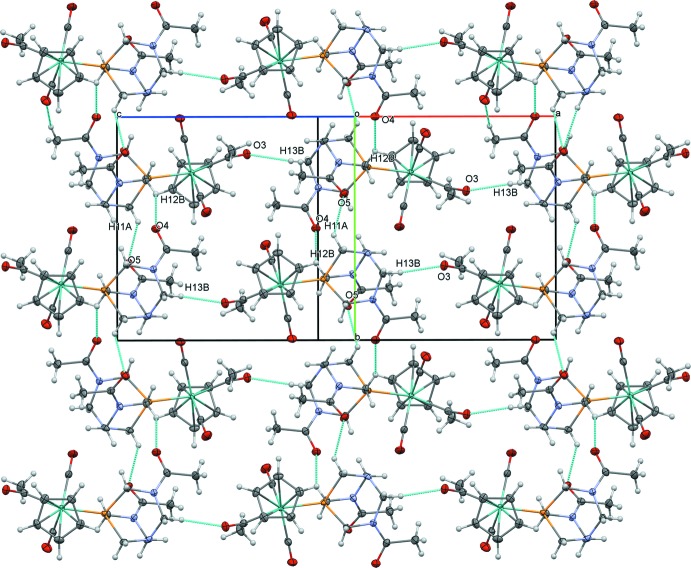
Layers of (2) formed by C—H⋯O inter­actions, viewed perpendicular to (101)

The acetamide groups of DAPTA, which are not present in PTA, play an important role in the extended structure of (2). Short, non-classical C—H⋯O inter­actions between O4 of an acetamide and H7 of a Cp ring (2.45 Å) link (2) into centrosymmetrical dimers (Table 4 ▸, Fig. 5 ▸). A combination of non-classical hydrogen bonds involving acetamide groups link (2) in chains along [010]: O5⋯H11*A* (2.41 Å) and O4⋯H12*B* (2.60 Å) (Fig. 6 ▸). These chains are further joined into layers parallel to (101) through the molybdenum acetyl group *via* a non-classical C—H⋯O inter­action between O3 and H13*B* (2.46 Å) (Fig. 7 ▸). The extensive network of inter­actions involving all three acetyl groups likely contribute to the low solubility of (2) in most organic solvents.

## Database survey   

The current version of the Cambridge Structural Database (Version 5.40, updated August 2019; Groom *et al.*, 2016 ▸) has thirteen entries corresponding to molybdenum acyl complexes of the general form Mo(C_5_H_5_)(CO)_2_(P*R*_3_)(CO*R*). The *trans*-dicarbonyl structure, as observed for (1) and (2), is preferred except in cases where the phosphine and acyl ligands are covalently linked, forcing them to be *cis* (Adams *et al.*, 1991 ▸; Mercier *et al.*, 1993 ▸; Yan *et al.*, 2009 ▸).

The PTA and DAPTA ligands have not been extensively utilized on molybdenum. There are eight instances of the PTA ligand bonded to molybdenum or tungsten, five of which involve coordination of one or more of the ligand to an *M*(CO)_*n*_ center to afford an octa­hedral product. Most relevant to this study is the tungsten complex W(C_5_H_5_)(CO)_2_(PTA)(H), which is analogous to (1) and (2) but features a hydride rather than an acyl ligand (Sears *et al.*, 2015 ▸).

## Synthesis and crystallization   

**CpMo(CO)_3_(CH_3_)**. This compound was prepared by a modification of the method used by Gladysz *et al.* (1979 ▸), as previously reported by Whited & Hofmeister (2014 ▸), with the modification that sodium tri­ethyl­borohydride (1.0 *M* in toluene) was used as a reductant instead of lithium tri­ethyl­borohydride to facilitate isolation of the product.

**CpMo(CO)_2_(PTA)(COCH_3_) (1)**. In an inert-atmosphere glove box, CpMo(CO)_3_(CH_3_) (325 mg, 1.25 mmol, 1.18 equivalents) was dissolved in aceto­nitrile (10 ml). In a separ­ate vial, 1,3,5-tri­aza-7-phosphaadamantane (PTA, 167 mg, 1.06 mmol, 1 equivalent) was massed. The homogeneous solution of the molybdenum complex was transferred to the vial containing PTA and the mixture was stirred at room temperature. After three days, the solution had generated a red solid that clung to the walls of the scintillation vial while the solution itself retained a red–orange color. Solvent was removed *in vacuo*, leaving a red–orange solid that was washed with hexa­nes (10 ml) and diethyl ether (10 ml) before a final extraction using tetra­hydro­furan (THF, 10 ml). The solid obtained from the THF fraction was the pale-yellow pure form of the final product (350 mg, 79%). Crystalline material was obtained as yellow–orange blocks by a THF/toluene vapor cross diffusion, which was used to concentrate the solution in a controlled manner without exposure to the glove-box atmosphere. The procedure is as follows: 50 mg of the product were dissolved in 1 ml of THF to form a concentrated solution. This solution was transferred into a small cylindrical 5 ml vial that was placed into a 20 ml vial. The remaining volume inside the 20 ml vial was filled with 10 ml of toluene. The vial was capped and left for two days before crystals were observed and harvested. ^1^H NMR (400 MHz, CDCl_3_): δ 5.16 (*d*, *J* = 1.2 Hz, 5H, C_5_ *H_5_*), 4.55 (*m*, 6H, P–C*H_2_*–N), 4.07 (*s*, 6H, N–C*H_2_*–N), 2.52 [*s*, 3H, C(O)C*H*_3_]. ^31^P{^1^H} NMR (162 MHz, CDCl_3_):δ −21.7 (*s*).

**CpMo(CO)_2_(DAPTA)(COCH_3_) (2)**. In an inert-atmos­phere glove box, CpMo(CO)_3_(CH_3_) (310 mg, 1.19 mmol, 1.05 equivalents) was dissolved in *N*,*N*-di­methyl­formamide (10 ml). In a separate vial, 3,7-diacetyl-1,3,5-tri­aza-5-phosphabi­cyclo­[3.3.1]nonane (DAPTA, 260 mg, 1.13 mmol, 1 equivalent) was massed. The homogeneous solution of the molybdenum complex was transferred to the vial containing DAPTA. The solution was not fully homogeneous, so vigorous stirring was employed. The solution had generated a pale-yellow solid after the first day while the supernatant solution was a red–orange color. The reaction mixture was filtered to obtain the pale-yellow solid, which was washed with two 2 ml portions of fresh *N*,*N*-di­methyl­formamide. The solid was dried *in vacuo* and recrystallized from a vapor diffusion of diethyl ether into a concentrated solution of the product in *N*,*N*-di­methyl­formamide, affording (2) as a pale-yellow crystalline material (80 mg, 15%). ^1^H NMR (400 MHz, DMSO-*d*_6_): δ 5.57 (*d*, *J* = 14 Hz, 1H), 5.36 (*s*, 5H, C_5_ *H_5_*), 5.27 (*dd*, *J*_PH_ = 6.8 Hz, *J*_HH_ = 15.2 Hz, 1H), 4.98 (*d*, *J*_HH_ = 14.0 Hz, 1H), 4.72 (*d*, *J*_HH_ = 14.0 Hz, 1H), 4.40 (*dd*, *J*_PH_ = 7.6 Hz, *J*_HH_ = 15.6 Hz, 1H), 4.20 (*d*, *J*_HH_ = 14 Hz, 1H), 4.05–3.98 (*m*, 1H), 3.75 (*s*, 2H), 3.46–3.39 (*m*, 1H), 2.42 (*s*, 3H, MoC(O)C*H*_3_), 1.98 (*s*, 3H, NC(O)C*H_3_*), 1.97 [*s*, 3H, NC(O)C*H_3_*]. ^31^P{^1^H} NMR (162 MHz, DMSO-*d*_6_):δ 8.06 (*s*).

## Refinement   

Crystal data, data collection and structure refinement details are summarized in Table 5 ▸. H atoms were placed in calculated positions and refined in the riding-model approximation with distances of C—H = 0.93, 0.96, and 0.97 Å for the cyclo­penta­dienyl, methyl, and methyl­ene groups, respectively, and with *U*_iso_(H) = *k*×*U*_eq_(C), *k* = 1.2 for cyclo­penta­dienyl and methyl­ene groups and 1.5 for methyl groups. Methyl group H atoms were allowed to rotate in order to find the best rotameric conformation.

A small number of intense low-angle reflections [one for (1); five for (2)] are missing from these high-quality data sets due to the arrangement of the instrument with a conservatively sized beam stop. The large number of reflections in the data sets (and the Fourier-transform relationship of intensities to atoms) ensures that no particular bias has been introduced.

## Supplementary Material

Crystal structure: contains datablock(s) 1, 2, general. DOI: 10.1107/S2056989020003679/zl2775sup1.cif

Structure factors: contains datablock(s) 1. DOI: 10.1107/S2056989020003679/zl27751sup2.hkl

Supporting information file. DOI: 10.1107/S2056989020003679/zl27751sup5.cdx

Structure factors: contains datablock(s) 2. DOI: 10.1107/S2056989020003679/zl27752sup3.hkl

Supporting information file. DOI: 10.1107/S2056989020003679/zl27752sup4.cdx

CCDC references: 1989805, 1989804

Additional supporting information:  crystallographic information; 3D view; checkCIF report

## Figures and Tables

**Figure 2:**
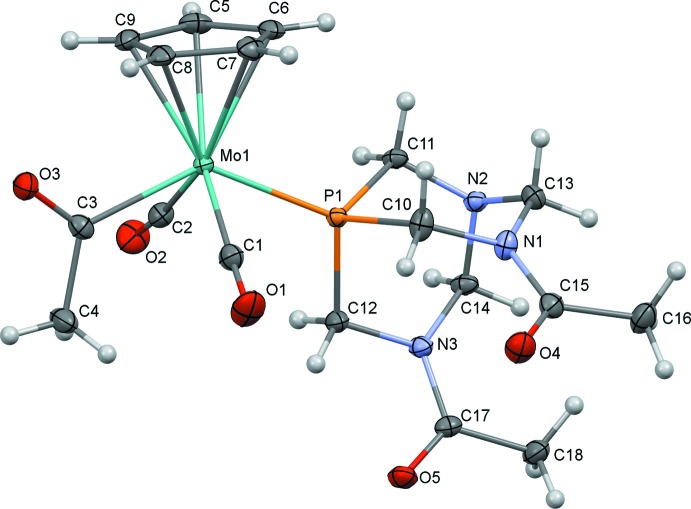
Mol­ecular structure of (2) with ellipsoids at 50% probability.

**Table 1:** Selected geometric parameters (Å, °) for (1)

Mo1—P1	2.4321 (5)	Mo1—C2	1.9746 (17)
Mo1—C1	1.9714 (17)	Mo1—C3	2.2398 (16)
			
C1—Mo1—P1	79.89 (5)	C2—Mo1—P1	78.80 (5)
C1—Mo1—C2	106.05 (7)	C2—Mo1—C3	78.51 (6)
C1—Mo1—C3	71.80 (6)	C3—Mo1—P1	136.77 (4)

**Table 2:** Selected geometric parameters (Å, °) for (2)

Mo1—P1	2.4258 (6)	Mo1—C2	1.971 (2)
Mo1—C1	1.964 (2)	Mo1—C3	2.243 (2)
			
C1—Mo1—P1	77.10 (7)	C2—Mo1—P1	81.75 (7)
C1—Mo1—C2	108.47 (10)	C2—Mo1—C3	76.10 (9)
C1—Mo1—C3	72.63 (9)	C3—Mo1—P1	133.99 (6)

**Table 3:** Hydrogen-bond geometry (Å, °) for (1)

*D*—H⋯*A*	*D*—H	H⋯*A*	*D*⋯*A*	*D*—H⋯*A*
C8—H8⋯O3^i^	0.95	2.49	3.269 (2)	139
C9—H9⋯O3^ii^	0.95	2.37	3.284 (2)	162

Symmetry codes: (i) 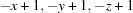; (ii) 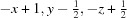.

**Table 4:** Hydrogen-bond geometry (Å, °) for (2)

*D*—H⋯*A*	*D*—H	H⋯*A*	*D*⋯*A*	*D*—H⋯*A*
C7—H7⋯O4^i^	0.93	2.45	3.353 (3)	163
C11—H11*A*⋯O5^ii^	0.97	2.41	3.211 (3)	140
C12—H12*B*⋯O4^ii^	0.97	2.60	3.409 (3)	141
C13—H13*A*⋯O5^iii^	0.97	2.57	3.474 (3)	156
C13—H13*B*⋯O3^iv^	0.97	2.46	3.261 (3)	139

Symmetry codes: (i) 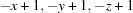; (ii) 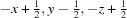; (iii) 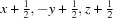; (iv) 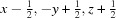.

**Table 5:** Experimental details

	(1)	(2)
Crystal data		
Chemical formula	[Mo(C_5_H_5_)(C_2_H_2_O)(C_6_H_12_N_3_P)(CO)_2_]	[Mo(C_5_H_5_)(C_2_H_3_O)(C_9_H_16_N_3_O_2_P)(CO)_2_]
*M*_r_	417.25	489.31
Crystal system, space group	Monoclinic, *P*2_1_/*c*	Monoclinic, *P*2_1_/*n*
Temperature (K)	100	100
*a*, *b*, *c* (Å)	9.7919 (14), 14.5757 (13), 12.1140 (12)	12.8674 (4), 11.6366 (3), 14.4655 (5)
β (°)	107.694 (6)	113.224 (4)
*V* (Å^3^)	1647.2 (3)	1990.45 (12)
*Z*	4	4
Radiation type	Mo *K*α	Mo *K*α
μ (mm^−1^)	0.91	0.77
Crystal size (mm)	0.2 × 0.19 × 0.15	0.17 × 0.1 × 0.02

Data collection		
Diffractometer	Bruker D8QUEST	Rigaku XtaLAB Synergy, Single source at offset/far, Pilatus 200K
Absorption correction	Multi-scan (*SADABS*; Bruker, 2016 ▸)	Multi-scan (*CrysAlis PRO*; Rigaku, 2019 ▸)
*T*_min_, *T*_max_	0.709, 0.746	0.868, 1.000
No. of measured, independent and observed [*I* > 2σ(*I*)] reflections	32227, 3785, 3379	38060, 4726, 4075
*R*_int_	0.040	0.044
(sin θ/λ)_max_ (Å^−1^)	0.651	0.658

Refinement		
*R*[*F* ^2^ > 2σ(*F* ^2^)], *wR*(*F* ^2^), *S*	0.020, 0.043, 1.07	0.028, 0.061, 1.09
No. of reflections	3785	4726
No. of parameters	210	256
H-atom treatment	H-atom parameters constrained	H-atom parameters constrained
Δρ_max_, Δρ_min_ (e Å^−3^)	0.45, −0.35	0.57, −0.37

Computer programs: *SAINT* (Bruker, 2018 ▸), *CrysAlis PRO* (Rigaku, 2019 ▸), *SHELXT2014/5* (Sheldrick, 2015*a* ▸), *SHELXL2018/3* (Sheldrick, 2015*b* ▸), *Mercury* (Macrae *et al.*, 2020 ▸) and *publCIF* (Westrip, 2010 ▸).

## supplementary crystallographic information

### trans-Acetyldicarbonyl(η5-cyclopentadienyl)(1,3,5-triaza-7-phosphaadamantane)molybdenum(II) (1) Crystal data

**Table:**

[Mo(C_5_H_5_)(C_2_H_2_O)(C_6_H_12_N_3_P)(CO)_2_]	*F*(000) = 848
*M**_r_* = 417.25	*D*_x_ = 1.683 Mg m^−^^3^
Monoclinic, *P*2_1_/*c*	Mo *K*α radiation, λ = 0.71073 Å
*a* = 9.7919 (14) Å	Cell parameters from 9370 reflections
*b* = 14.5757 (13) Å	θ = 3.3–27.5°
*c* = 12.1140 (12) Å	µ = 0.91 mm^−^^1^
β = 107.694 (6)°	*T* = 100 K
*V* = 1647.2 (3) Å^3^	Block, orange
*Z* = 4	0.2 × 0.19 × 0.15 mm

### trans-Acetyldicarbonyl(η5-cyclopentadienyl)(1,3,5-triaza-7-phosphaadamantane)molybdenum(II) (1) Data collection

**Table:**

Bruker D8QUEST diffractometer	3379 reflections with *I* > 2σ(*I*)
ω and φ scans	*R*_int_ = 0.040
Absorption correction: multi-scan (SADABS; Bruker, 2016)	θ_max_ = 27.6°, θ_min_ = 3.3°
*T*_min_ = 0.709, *T*_max_ = 0.746	*h* = −12→12
32227 measured reflections	*k* = −18→18
3785 independent reflections	*l* = −15→15

### trans-Acetyldicarbonyl(η5-cyclopentadienyl)(1,3,5-triaza-7-phosphaadamantane)molybdenum(II) (1) Refinement

**Table:**

Refinement on *F*^2^	Hydrogen site location: inferred from neighbouring sites
Least-squares matrix: full	H-atom parameters constrained
*R*[*F*^2^ > 2σ(*F*^2^)] = 0.020	*w* = 1/[σ^2^(*F*_o_^2^) + (0.0124*P*)^2^ + 1.3596*P*] where *P* = (*F*_o_^2^ + 2*F*_c_^2^)/3
*wR*(*F*^2^) = 0.043	(Δ/σ)_max_ = 0.001
*S* = 1.07	Δρ_max_ = 0.45 e Å^−^^3^
3785 reflections	Δρ_min_ = −0.35 e Å^−^^3^
210 parameters	Extinction correction: SHELXL2018/3 (Sheldrick 2015b), Fc^*^=kFc[1+0.001xFc^2^λ^3^/sin(2θ)]^-1/4^
0 restraints	Extinction coefficient: 0.0040 (2)
Primary atom site location: dual	

### trans-Acetyldicarbonyl(η5-cyclopentadienyl)(1,3,5-triaza-7-phosphaadamantane)molybdenum(II) (1) Special details

**Table:**

Geometry. All esds (except the esd in the dihedral angle between two l.s. planes) are estimated using the full covariance matrix. The cell esds are taken into account individually in the estimation of esds in distances, angles and torsion angles; correlations between esds in cell parameters are only used when they are defined by crystal symmetry. An approximate (isotropic) treatment of cell esds is used for estimating esds involving l.s. planes.

### trans-Acetyldicarbonyl(η5-cyclopentadienyl)(1,3,5-triaza-7-phosphaadamantane)molybdenum(II) (1) Fractional atomic coordinates and isotropic or equivalent isotropic displacement parameters (Å^2^)

**Table:**

	*x*	*y*	*z*	*U*_iso_*/*U*_eq_	
Mo1	0.59777 (2)	0.49328 (2)	0.28768 (2)	0.00886 (5)	
P1	0.76523 (4)	0.41400 (3)	0.20808 (4)	0.01095 (9)	
O1	0.65998 (14)	0.65083 (9)	0.13519 (11)	0.0225 (3)	
O2	0.85977 (13)	0.47150 (9)	0.51067 (11)	0.0203 (3)	
O3	0.47562 (13)	0.66109 (8)	0.37491 (10)	0.0174 (3)	
N1	0.81148 (15)	0.31674 (11)	0.02647 (13)	0.0187 (3)	
N2	0.95915 (17)	0.27288 (11)	0.22389 (14)	0.0216 (3)	
N3	1.00813 (15)	0.41777 (10)	0.13718 (13)	0.0172 (3)	
C1	0.64170 (17)	0.59232 (12)	0.19293 (14)	0.0145 (3)	
C2	0.76722 (17)	0.48263 (11)	0.42615 (14)	0.0131 (3)	
C3	0.59049 (18)	0.62704 (11)	0.37674 (13)	0.0126 (3)	
C4	0.72533 (19)	0.67895 (11)	0.44048 (15)	0.0172 (3)	
H4A	0.768353	0.651212	0.516872	0.026*	
H4B	0.701269	0.743146	0.449920	0.026*	
H4C	0.793647	0.676106	0.395903	0.026*	
C5	0.39034 (18)	0.42909 (12)	0.16033 (15)	0.0165 (3)	
H5	0.372255	0.432040	0.078793	0.020*	
C6	0.35024 (17)	0.49564 (12)	0.22997 (14)	0.0155 (3)	
H6	0.300765	0.551374	0.203612	0.019*	
C7	0.39757 (17)	0.46382 (12)	0.34674 (15)	0.0155 (3)	
H7	0.385727	0.495068	0.412043	0.019*	
C8	0.46496 (18)	0.37807 (12)	0.34879 (15)	0.0164 (3)	
H8	0.504922	0.340967	0.415265	0.020*	
C9	0.46240 (18)	0.35724 (11)	0.23437 (15)	0.0164 (3)	
H9	0.502247	0.304029	0.210865	0.020*	
C10	0.69704 (17)	0.36231 (12)	0.06169 (14)	0.0155 (3)	
H10A	0.621580	0.316952	0.061143	0.019*	
H10B	0.653123	0.410872	0.004845	0.019*	
C11	0.86374 (19)	0.31312 (12)	0.28477 (16)	0.0196 (4)	
H11A	0.921558	0.331544	0.363817	0.023*	
H11B	0.794116	0.266087	0.292213	0.023*	
C12	0.91942 (17)	0.47689 (12)	0.18647 (15)	0.0163 (3)	
H12A	0.883561	0.529431	0.133844	0.020*	
H12B	0.979281	0.501540	0.261788	0.020*	
C13	0.8764 (2)	0.24240 (13)	0.10693 (17)	0.0232 (4)	
H13A	0.940468	0.206656	0.073719	0.028*	
H13B	0.799353	0.200736	0.113403	0.028*	
C14	1.06640 (18)	0.34022 (13)	0.21383 (16)	0.0220 (4)	
H14A	1.116912	0.364261	0.292037	0.026*	
H14B	1.138175	0.308452	0.184965	0.026*	
C15	0.92521 (19)	0.38166 (13)	0.02367 (15)	0.0202 (4)	
H15A	0.991757	0.350678	−0.011587	0.024*	
H15B	0.881223	0.433817	−0.027049	0.024*	

### trans-Acetyldicarbonyl(η5-cyclopentadienyl)(1,3,5-triaza-7-phosphaadamantane)molybdenum(II) (1) Atomic displacement parameters (Å^2^)

**Table:**

	*U*^11^	*U*^22^	*U*^33^	*U*^12^	*U*^13^	*U*^23^
Mo1	0.00893 (7)	0.00963 (7)	0.00871 (7)	−0.00047 (5)	0.00374 (5)	0.00005 (5)
P1	0.01048 (19)	0.0127 (2)	0.01055 (19)	0.00010 (15)	0.00453 (15)	−0.00002 (15)
O1	0.0294 (7)	0.0204 (7)	0.0202 (7)	−0.0010 (5)	0.0112 (6)	0.0064 (5)
O2	0.0154 (6)	0.0258 (7)	0.0171 (6)	0.0012 (5)	0.0009 (5)	0.0017 (5)
O3	0.0188 (6)	0.0139 (6)	0.0211 (6)	0.0019 (5)	0.0087 (5)	−0.0011 (5)
N1	0.0138 (7)	0.0263 (8)	0.0182 (7)	−0.0026 (6)	0.0081 (6)	−0.0083 (6)
N2	0.0202 (7)	0.0230 (8)	0.0251 (8)	0.0084 (6)	0.0119 (7)	0.0022 (6)
N3	0.0120 (7)	0.0238 (8)	0.0176 (7)	−0.0007 (6)	0.0069 (6)	−0.0027 (6)
C1	0.0131 (8)	0.0179 (8)	0.0128 (8)	0.0013 (6)	0.0044 (6)	−0.0018 (6)
C2	0.0141 (8)	0.0124 (8)	0.0153 (8)	−0.0004 (6)	0.0082 (7)	−0.0005 (6)
C3	0.0193 (8)	0.0100 (7)	0.0094 (7)	0.0006 (6)	0.0057 (6)	0.0025 (6)
C4	0.0207 (9)	0.0139 (8)	0.0161 (8)	−0.0017 (7)	0.0042 (7)	0.0000 (6)
C5	0.0139 (8)	0.0212 (9)	0.0136 (8)	−0.0057 (7)	0.0027 (7)	−0.0039 (7)
C6	0.0083 (7)	0.0182 (8)	0.0188 (8)	−0.0017 (6)	0.0023 (6)	−0.0021 (7)
C7	0.0124 (8)	0.0205 (8)	0.0169 (8)	−0.0058 (6)	0.0094 (7)	−0.0049 (7)
C8	0.0164 (8)	0.0168 (8)	0.0170 (8)	−0.0062 (6)	0.0066 (7)	0.0021 (6)
C9	0.0167 (8)	0.0128 (8)	0.0226 (9)	−0.0057 (6)	0.0101 (7)	−0.0042 (7)
C10	0.0105 (7)	0.0222 (9)	0.0146 (8)	−0.0018 (6)	0.0051 (6)	−0.0047 (7)
C11	0.0217 (9)	0.0209 (9)	0.0190 (9)	0.0089 (7)	0.0105 (7)	0.0058 (7)
C12	0.0127 (8)	0.0181 (8)	0.0197 (8)	−0.0029 (6)	0.0071 (7)	−0.0043 (6)
C13	0.0218 (9)	0.0191 (9)	0.0328 (10)	0.0005 (7)	0.0143 (8)	−0.0081 (8)
C14	0.0126 (8)	0.0319 (10)	0.0212 (9)	0.0048 (7)	0.0047 (7)	−0.0025 (8)
C15	0.0154 (8)	0.0306 (10)	0.0173 (9)	−0.0029 (7)	0.0090 (7)	−0.0036 (7)

### trans-Acetyldicarbonyl(η5-cyclopentadienyl)(1,3,5-triaza-7-phosphaadamantane)molybdenum(II) (1) Geometric parameters (Å, º)

**Table:**

Mo1—P1	2.4321 (5)	C4—H4A	0.9800
Mo1—C1	1.9714 (17)	C4—H4B	0.9800
Mo1—C2	1.9746 (17)	C4—H4C	0.9800
Mo1—C3	2.2398 (16)	C5—H5	0.9500
Mo1—C5	2.3401 (16)	C5—C6	1.417 (2)
Mo1—C6	2.3097 (16)	C5—C9	1.419 (2)
Mo1—C7	2.3221 (16)	C6—H6	0.9500
Mo1—C8	2.3759 (16)	C6—C7	1.426 (2)
Mo1—C9	2.3634 (16)	C7—H7	0.9500
P1—C10	1.8543 (17)	C7—C8	1.410 (2)
P1—C11	1.8468 (17)	C8—H8	0.9500
P1—C12	1.8517 (17)	C8—C9	1.412 (2)
O1—C1	1.151 (2)	C9—H9	0.9500
O2—C2	1.154 (2)	C10—H10A	0.9900
O3—C3	1.223 (2)	C10—H10B	0.9900
N1—C10	1.473 (2)	C11—H11A	0.9900
N1—C13	1.467 (2)	C11—H11B	0.9900
N1—C15	1.470 (2)	C12—H12A	0.9900
N2—C11	1.476 (2)	C12—H12B	0.9900
N2—C13	1.471 (2)	C13—H13A	0.9900
N2—C14	1.469 (2)	C13—H13B	0.9900
N3—C12	1.473 (2)	C14—H14A	0.9900
N3—C14	1.464 (2)	C14—H14B	0.9900
N3—C15	1.466 (2)	C15—H15A	0.9900
C3—C4	1.514 (2)	C15—H15B	0.9900
			
C1—Mo1—P1	79.89 (5)	C6—C5—H5	126.2
C1—Mo1—C2	106.05 (7)	C6—C5—C9	107.66 (15)
C1—Mo1—C3	71.80 (6)	C9—C5—Mo1	73.34 (9)
C1—Mo1—C5	102.02 (6)	C9—C5—H5	126.2
C1—Mo1—C6	101.98 (6)	Mo1—C6—H6	119.7
C1—Mo1—C7	131.98 (6)	C5—C6—Mo1	73.43 (9)
C1—Mo1—C8	159.38 (6)	C5—C6—H6	126.2
C1—Mo1—C9	131.21 (6)	C5—C6—C7	107.62 (15)
C2—Mo1—P1	78.80 (5)	C7—C6—Mo1	72.55 (9)
C2—Mo1—C3	78.51 (6)	C7—C6—H6	126.2
C2—Mo1—C5	149.93 (6)	Mo1—C7—H7	119.7
C2—Mo1—C6	142.35 (6)	C6—C7—Mo1	71.60 (9)
C2—Mo1—C7	107.08 (6)	C6—C7—H7	125.8
C2—Mo1—C8	94.47 (6)	C8—C7—Mo1	74.63 (9)
C2—Mo1—C9	115.01 (6)	C8—C7—C6	108.37 (15)
C3—Mo1—P1	136.77 (4)	C8—C7—H7	125.8
C3—Mo1—C5	121.15 (6)	Mo1—C8—H8	122.9
C3—Mo1—C6	87.06 (6)	C7—C8—Mo1	70.46 (9)
C3—Mo1—C7	81.90 (6)	C7—C8—H8	126.1
C3—Mo1—C8	111.38 (6)	C7—C8—C9	107.74 (15)
C3—Mo1—C9	139.87 (6)	C9—C8—Mo1	72.19 (9)
C5—Mo1—P1	95.93 (4)	C9—C8—H8	126.1
C5—Mo1—C8	58.34 (6)	Mo1—C9—H9	121.3
C5—Mo1—C9	35.12 (6)	C5—C9—Mo1	71.54 (9)
C6—Mo1—P1	131.22 (4)	C5—C9—H9	125.7
C6—Mo1—C5	35.48 (6)	C8—C9—Mo1	73.15 (9)
C6—Mo1—C7	35.85 (6)	C8—C9—C5	108.60 (15)
C6—Mo1—C8	58.76 (6)	C8—C9—H9	125.7
C6—Mo1—C9	58.66 (6)	P1—C10—H10A	109.2
C7—Mo1—P1	140.20 (4)	P1—C10—H10B	109.2
C7—Mo1—C5	58.95 (6)	N1—C10—P1	112.07 (11)
C7—Mo1—C8	34.91 (6)	N1—C10—H10A	109.2
C7—Mo1—C9	58.20 (6)	N1—C10—H10B	109.2
C8—Mo1—P1	106.65 (4)	H10A—C10—H10B	107.9
C9—Mo1—P1	83.27 (4)	P1—C11—H11A	109.2
C9—Mo1—C8	34.66 (6)	P1—C11—H11B	109.2
C10—P1—Mo1	118.82 (5)	N2—C11—P1	112.21 (12)
C11—P1—Mo1	119.40 (6)	N2—C11—H11A	109.2
C11—P1—C10	97.98 (8)	N2—C11—H11B	109.2
C11—P1—C12	98.14 (8)	H11A—C11—H11B	107.9
C12—P1—Mo1	119.85 (6)	P1—C12—H12A	109.2
C12—P1—C10	97.93 (8)	P1—C12—H12B	109.2
C13—N1—C10	110.79 (14)	N3—C12—P1	111.98 (11)
C13—N1—C15	108.21 (14)	N3—C12—H12A	109.2
C15—N1—C10	111.40 (14)	N3—C12—H12B	109.2
C13—N2—C11	110.75 (14)	H12A—C12—H12B	107.9
C14—N2—C11	110.66 (14)	N1—C13—N2	114.68 (15)
C14—N2—C13	108.68 (14)	N1—C13—H13A	108.6
C14—N3—C12	111.03 (14)	N1—C13—H13B	108.6
C14—N3—C15	108.41 (14)	N2—C13—H13A	108.6
C15—N3—C12	111.33 (13)	N2—C13—H13B	108.6
O1—C1—Mo1	176.52 (15)	H13A—C13—H13B	107.6
O2—C2—Mo1	174.40 (14)	N2—C14—H14A	108.6
O3—C3—Mo1	120.54 (12)	N2—C14—H14B	108.6
O3—C3—C4	117.45 (15)	N3—C14—N2	114.69 (14)
C4—C3—Mo1	122.01 (11)	N3—C14—H14A	108.6
C3—C4—H4A	109.5	N3—C14—H14B	108.6
C3—C4—H4B	109.5	H14A—C14—H14B	107.6
C3—C4—H4C	109.5	N1—C15—H15A	108.6
H4A—C4—H4B	109.5	N1—C15—H15B	108.6
H4A—C4—H4C	109.5	N3—C15—N1	114.65 (14)
H4B—C4—H4C	109.5	N3—C15—H15A	108.6
Mo1—C5—H5	121.2	N3—C15—H15B	108.6
C6—C5—Mo1	71.09 (9)	H15A—C15—H15B	107.6
			
Mo1—P1—C10—N1	−179.79 (10)	C10—N1—C15—N3	66.86 (19)
Mo1—P1—C11—N2	179.31 (10)	C11—P1—C10—N1	−49.79 (14)
Mo1—P1—C12—N3	−179.75 (9)	C11—P1—C12—N3	49.41 (13)
Mo1—C5—C6—C7	−64.99 (11)	C11—N2—C13—N1	67.75 (19)
Mo1—C5—C9—C8	64.24 (12)	C11—N2—C14—N3	−67.81 (19)
Mo1—C6—C7—C8	−66.13 (11)	C12—P1—C10—N1	49.65 (14)
Mo1—C7—C8—C9	−62.98 (11)	C12—P1—C11—N2	−49.56 (14)
Mo1—C8—C9—C5	−63.20 (11)	C12—N3—C14—N2	68.04 (18)
C5—C6—C7—Mo1	65.58 (11)	C12—N3—C15—N1	−67.16 (19)
C5—C6—C7—C8	−0.55 (18)	C13—N1—C10—P1	60.54 (16)
C6—C5—C9—Mo1	−63.24 (11)	C13—N1—C15—N3	−55.17 (19)
C6—C5—C9—C8	1.00 (18)	C13—N2—C11—P1	−60.33 (18)
C6—C7—C8—Mo1	64.14 (11)	C13—N2—C14—N3	54.00 (19)
C6—C7—C8—C9	1.17 (18)	C14—N2—C11—P1	60.25 (17)
C7—C8—C9—Mo1	61.86 (11)	C14—N2—C13—N1	−54.01 (19)
C7—C8—C9—C5	−1.34 (18)	C14—N3—C12—P1	−60.30 (16)
C9—C5—C6—Mo1	64.71 (11)	C14—N3—C15—N1	55.25 (19)
C9—C5—C6—C7	−0.28 (18)	C15—N1—C10—P1	−59.98 (17)
C10—P1—C11—N2	49.70 (14)	C15—N1—C13—N2	54.43 (19)
C10—P1—C12—N3	−49.88 (13)	C15—N3—C12—P1	60.59 (17)
C10—N1—C13—N2	−67.97 (19)	C15—N3—C14—N2	−54.55 (19)

### trans-Acetyldicarbonyl(η5-cyclopentadienyl)(1,3,5-triaza-7-phosphaadamantane)molybdenum(II) (1) Hydrogen-bond geometry (Å, º)

**Table:**

*D*—H···*A*	*D*—H	H···*A*	*D*···*A*	*D*—H···*A*
C8—H8···O3^i^	0.95	2.49	3.269 (2)	139
C9—H9···O3^ii^	0.95	2.37	3.284 (2)	162

Symmetry codes: (i) −*x*+1, −*y*+1, −*z*+1; (ii) −*x*+1, *y*−1/2, −*z*+1/2.

### trans-Acetyldicarbonyl(η5-cyclopentadienyl)(3,7-diacetyl-1,3,7-triaza-5-phosphabicyclo[3.3.1]nonane)molybdenum(II) (2) Crystal data

**Table:**

[Mo(C_5_H_5_)(C_2_H_3_O)(C_9_H_16_N_3_O_2_P)(CO)_2_]	*F*(000) = 1000
*M**_r_* = 489.31	*D*_x_ = 1.633 Mg m^−^^3^
Monoclinic, *P*2_1_/*n*	Mo *K*α radiation, λ = 0.71073 Å
*a* = 12.8674 (4) Å	Cell parameters from 13952 reflections
*b* = 11.6366 (3) Å	θ = 2.5–31.0°
*c* = 14.4655 (5) Å	µ = 0.77 mm^−^^1^
β = 113.224 (4)°	*T* = 100 K
*V* = 1990.45 (12) Å^3^	Plate, clear colorless
*Z* = 4	0.17 × 0.1 × 0.02 mm

### trans-Acetyldicarbonyl(η5-cyclopentadienyl)(3,7-diacetyl-1,3,7-triaza-5-phosphabicyclo[3.3.1]nonane)molybdenum(II) (2) Data collection

**Table:**

Rigaku XtaLAB Synergy, Single source at offset/far, Pilatus 200K diffractometer	4726 independent reflections
Radiation source: micro-focus sealed X-ray tube, PhotonJet (Mo) X-ray Source	4075 reflections with *I* > 2σ(*I*)
Mirror monochromator	*R*_int_ = 0.044
Detector resolution: 5.8140 pixels mm^-1^	θ_max_ = 27.9°, θ_min_ = 2.3°
ω scans	*h* = −16→16
Absorption correction: multi-scan (CrysAlisPro; Rigaku, 2019)	*k* = −15→15
*T*_min_ = 0.868, *T*_max_ = 1.000	*l* = −19→19
38060 measured reflections	

### trans-Acetyldicarbonyl(η5-cyclopentadienyl)(3,7-diacetyl-1,3,7-triaza-5-phosphabicyclo[3.3.1]nonane)molybdenum(II) (2) Refinement

**Table:**

Refinement on *F*^2^	Primary atom site location: dual
Least-squares matrix: full	Secondary atom site location: difference Fourier map
*R*[*F*^2^ > 2σ(*F*^2^)] = 0.028	Hydrogen site location: inferred from neighbouring sites
*wR*(*F*^2^) = 0.061	H-atom parameters constrained
*S* = 1.09	*w* = 1/[σ^2^(*F*_o_^2^) + (0.0017*P*)^2^ + 3.9699*P*] where *P* = (*F*_o_^2^ + 2*F*_c_^2^)/3
4726 reflections	(Δ/σ)_max_ = 0.002
256 parameters	Δρ_max_ = 0.57 e Å^−^^3^
0 restraints	Δρ_min_ = −0.37 e Å^−^^3^

### trans-Acetyldicarbonyl(η5-cyclopentadienyl)(3,7-diacetyl-1,3,7-triaza-5-phosphabicyclo[3.3.1]nonane)molybdenum(II) (2) Special details

**Table:**

Geometry. All esds (except the esd in the dihedral angle between two l.s. planes) are estimated using the full covariance matrix. The cell esds are taken into account individually in the estimation of esds in distances, angles and torsion angles; correlations between esds in cell parameters are only used when they are defined by crystal symmetry. An approximate (isotropic) treatment of cell esds is used for estimating esds involving l.s. planes.

### trans-Acetyldicarbonyl(η5-cyclopentadienyl)(3,7-diacetyl-1,3,7-triaza-5-phosphabicyclo[3.3.1]nonane)molybdenum(II) (2) Fractional atomic coordinates and isotropic or equivalent isotropic displacement parameters (Å^2^)

**Table:**

	*x*	*y*	*z*	*U*_iso_*/*U*_eq_	
Mo1	0.63575 (2)	0.25030 (2)	0.30169 (2)	0.01028 (5)	
P1	0.47121 (4)	0.22642 (5)	0.34228 (4)	0.01101 (11)	
O1	0.52463 (17)	0.49328 (16)	0.25544 (15)	0.0312 (4)	
O2	0.50920 (15)	0.07353 (16)	0.13206 (13)	0.0270 (4)	
O3	0.75732 (14)	0.33298 (15)	0.17091 (12)	0.0209 (4)	
O4	0.30320 (15)	0.49974 (15)	0.41980 (13)	0.0242 (4)	
O5	0.14095 (13)	0.34628 (14)	0.16401 (12)	0.0195 (3)	
N1	0.36745 (16)	0.31852 (17)	0.46343 (14)	0.0165 (4)	
N2	0.35305 (15)	0.11049 (16)	0.43014 (13)	0.0135 (4)	
N3	0.23918 (15)	0.20618 (16)	0.27035 (14)	0.0147 (4)	
C1	0.5628 (2)	0.4017 (2)	0.26999 (18)	0.0185 (5)	
C2	0.55033 (19)	0.1431 (2)	0.19267 (17)	0.0173 (5)	
C3	0.66223 (19)	0.3187 (2)	0.16760 (16)	0.0163 (4)	
C4	0.5638 (2)	0.3496 (2)	0.06996 (18)	0.0239 (5)	
H4A	0.519961	0.281951	0.041945	0.036*	
H4B	0.592380	0.380455	0.023061	0.036*	
H4C	0.516914	0.405872	0.083315	0.036*	
C5	0.76382 (19)	0.1081 (2)	0.39905 (17)	0.0197 (5)	
H5	0.748692	0.030017	0.387962	0.024*	
C6	0.73813 (19)	0.1777 (2)	0.46709 (16)	0.0179 (5)	
H6	0.702176	0.153239	0.508153	0.022*	
C7	0.77602 (19)	0.2908 (2)	0.46259 (17)	0.0184 (5)	
H7	0.770231	0.353153	0.500496	0.022*	
C8	0.82445 (18)	0.2920 (2)	0.38987 (17)	0.0188 (5)	
H8	0.855613	0.355473	0.371134	0.023*	
C9	0.81675 (19)	0.1787 (2)	0.35077 (17)	0.0192 (5)	
H9	0.842145	0.155039	0.301873	0.023*	
C10	0.4569 (2)	0.3377 (2)	0.42664 (18)	0.0184 (5)	
H10A	0.528327	0.344180	0.484207	0.022*	
H10B	0.442864	0.410721	0.391445	0.022*	
C11	0.45578 (18)	0.09925 (19)	0.41018 (16)	0.0135 (4)	
H11A	0.450942	0.030898	0.370298	0.016*	
H11B	0.521186	0.091764	0.473140	0.016*	
C12	0.33213 (18)	0.2198 (2)	0.23700 (17)	0.0168 (5)	
H12A	0.320423	0.289683	0.197633	0.020*	
H12B	0.331263	0.155760	0.193769	0.020*	
C13	0.3622 (2)	0.2021 (2)	0.50113 (17)	0.0170 (5)	
H13A	0.429693	0.189409	0.561541	0.020*	
H13B	0.297707	0.197899	0.519767	0.020*	
C14	0.25059 (18)	0.10856 (19)	0.33896 (17)	0.0151 (4)	
H14A	0.185886	0.108202	0.357482	0.018*	
H14B	0.248845	0.037642	0.303134	0.018*	
C15	0.29827 (19)	0.4079 (2)	0.46058 (17)	0.0176 (5)	
C16	0.2176 (2)	0.3947 (2)	0.5127 (2)	0.0250 (5)	
H16A	0.259315	0.397149	0.584258	0.037*	
H16B	0.178938	0.322462	0.494274	0.037*	
H16C	0.163490	0.456177	0.492731	0.037*	
C17	0.14790 (18)	0.27602 (19)	0.22978 (16)	0.0152 (4)	
C18	0.05350 (19)	0.2655 (2)	0.26638 (18)	0.0216 (5)	
H18A	0.010423	0.335387	0.252252	0.032*	
H18B	0.084905	0.251691	0.337578	0.032*	
H18C	0.005112	0.202610	0.232574	0.032*	

### trans-Acetyldicarbonyl(η5-cyclopentadienyl)(3,7-diacetyl-1,3,7-triaza-5-phosphabicyclo[3.3.1]nonane)molybdenum(II) (2) Atomic displacement parameters (Å^2^)

**Table:**

	*U*^11^	*U*^22^	*U*^33^	*U*^12^	*U*^13^	*U*^23^
Mo1	0.00936 (8)	0.01073 (9)	0.01029 (8)	0.00077 (7)	0.00339 (6)	0.00039 (7)
P1	0.0108 (2)	0.0103 (3)	0.0115 (2)	0.00093 (19)	0.0039 (2)	0.00144 (19)
O1	0.0374 (11)	0.0174 (9)	0.0469 (12)	0.0094 (8)	0.0253 (10)	0.0102 (8)
O2	0.0255 (9)	0.0273 (10)	0.0249 (9)	−0.0042 (8)	0.0063 (8)	−0.0112 (8)
O3	0.0181 (8)	0.0268 (9)	0.0193 (8)	−0.0005 (7)	0.0088 (7)	0.0017 (7)
O4	0.0283 (9)	0.0172 (9)	0.0308 (10)	0.0027 (7)	0.0158 (8)	0.0035 (7)
O5	0.0175 (8)	0.0192 (9)	0.0203 (8)	0.0031 (7)	0.0059 (7)	0.0087 (7)
N1	0.0185 (9)	0.0144 (10)	0.0204 (10)	−0.0016 (7)	0.0117 (8)	−0.0020 (7)
N2	0.0118 (9)	0.0135 (9)	0.0143 (9)	0.0015 (7)	0.0042 (7)	0.0042 (7)
N3	0.0113 (8)	0.0164 (9)	0.0155 (9)	0.0001 (7)	0.0041 (7)	0.0037 (7)
C1	0.0183 (11)	0.0194 (12)	0.0197 (11)	−0.0009 (9)	0.0096 (9)	0.0032 (9)
C2	0.0144 (10)	0.0199 (12)	0.0179 (11)	0.0017 (9)	0.0066 (9)	0.0013 (9)
C3	0.0192 (11)	0.0156 (11)	0.0136 (10)	−0.0007 (9)	0.0060 (9)	−0.0003 (8)
C4	0.0195 (12)	0.0338 (14)	0.0164 (11)	−0.0006 (10)	0.0048 (10)	0.0068 (10)
C5	0.0172 (11)	0.0161 (12)	0.0193 (11)	0.0047 (9)	0.0002 (9)	0.0023 (9)
C6	0.0122 (10)	0.0251 (12)	0.0126 (10)	0.0029 (9)	0.0008 (8)	0.0057 (9)
C7	0.0170 (11)	0.0209 (12)	0.0132 (10)	0.0007 (9)	0.0016 (9)	−0.0031 (9)
C8	0.0101 (10)	0.0258 (12)	0.0164 (11)	−0.0005 (9)	0.0008 (9)	0.0027 (9)
C9	0.0120 (10)	0.0280 (13)	0.0154 (11)	0.0058 (9)	0.0031 (9)	−0.0007 (9)
C10	0.0219 (12)	0.0134 (11)	0.0248 (12)	−0.0041 (9)	0.0144 (10)	−0.0041 (9)
C11	0.0122 (10)	0.0121 (10)	0.0146 (10)	0.0015 (8)	0.0037 (8)	0.0032 (8)
C12	0.0127 (10)	0.0217 (12)	0.0152 (10)	0.0020 (8)	0.0048 (8)	0.0041 (8)
C13	0.0183 (11)	0.0187 (11)	0.0154 (11)	0.0013 (9)	0.0081 (9)	0.0032 (9)
C14	0.0120 (10)	0.0131 (11)	0.0179 (11)	−0.0006 (8)	0.0034 (9)	0.0038 (8)
C15	0.0177 (11)	0.0194 (12)	0.0147 (10)	−0.0006 (9)	0.0054 (9)	−0.0025 (9)
C16	0.0245 (13)	0.0229 (13)	0.0336 (14)	0.0066 (10)	0.0180 (11)	0.0043 (10)
C17	0.0122 (10)	0.0151 (11)	0.0150 (10)	0.0003 (8)	0.0019 (8)	−0.0007 (8)
C18	0.0156 (10)	0.0251 (13)	0.0252 (12)	0.0051 (10)	0.0091 (9)	0.0079 (10)

### trans-Acetyldicarbonyl(η5-cyclopentadienyl)(3,7-diacetyl-1,3,7-triaza-5-phosphabicyclo[3.3.1]nonane)molybdenum(II) (2) Geometric parameters (Å, º)

**Table:**

Mo1—P1	2.4258 (6)	C4—H4C	0.9600
Mo1—C1	1.964 (2)	C5—H5	0.9300
Mo1—C2	1.971 (2)	C5—C6	1.411 (3)
Mo1—C3	2.243 (2)	C5—C9	1.415 (3)
Mo1—C5	2.368 (2)	C6—H6	0.9300
Mo1—C6	2.385 (2)	C6—C7	1.413 (3)
Mo1—C7	2.363 (2)	C7—H7	0.9300
Mo1—C8	2.306 (2)	C7—C8	1.419 (3)
Mo1—C9	2.306 (2)	C8—H8	0.9300
P1—C10	1.838 (2)	C8—C9	1.423 (3)
P1—C11	1.830 (2)	C9—H9	0.9300
P1—C12	1.838 (2)	C10—H10A	0.9700
O1—C1	1.157 (3)	C10—H10B	0.9700
O2—C2	1.157 (3)	C11—H11A	0.9700
O3—C3	1.217 (3)	C11—H11B	0.9700
O4—C15	1.234 (3)	C12—H12A	0.9700
O5—C17	1.231 (3)	C12—H12B	0.9700
N1—C10	1.463 (3)	C13—H13A	0.9700
N1—C13	1.471 (3)	C13—H13B	0.9700
N1—C15	1.359 (3)	C14—H14A	0.9700
N2—C11	1.466 (3)	C14—H14B	0.9700
N2—C13	1.453 (3)	C15—C16	1.512 (3)
N2—C14	1.451 (3)	C16—H16A	0.9600
N3—C12	1.464 (3)	C16—H16B	0.9600
N3—C14	1.477 (3)	C16—H16C	0.9600
N3—C17	1.357 (3)	C17—C18	1.509 (3)
C3—C4	1.522 (3)	C18—H18A	0.9600
C4—H4A	0.9600	C18—H18B	0.9600
C4—H4B	0.9600	C18—H18C	0.9600
			
C1—Mo1—P1	77.10 (7)	C5—C6—H6	125.6
C1—Mo1—C2	108.47 (10)	C5—C6—C7	108.8 (2)
C1—Mo1—C3	72.63 (9)	C7—C6—Mo1	71.84 (13)
C1—Mo1—C5	157.27 (9)	C7—C6—H6	125.6
C1—Mo1—C6	125.17 (9)	Mo1—C7—H7	121.9
C1—Mo1—C7	99.22 (9)	C6—C7—Mo1	73.52 (13)
C1—Mo1—C8	104.03 (9)	C6—C7—H7	126.2
C1—Mo1—C9	136.72 (9)	C6—C7—C8	107.7 (2)
C2—Mo1—P1	81.75 (7)	C8—C7—Mo1	70.12 (12)
C2—Mo1—C3	76.10 (9)	C8—C7—H7	126.2
C2—Mo1—C5	94.08 (9)	Mo1—C8—H8	119.1
C2—Mo1—C6	119.45 (9)	C7—C8—Mo1	74.54 (13)
C2—Mo1—C7	151.96 (9)	C7—C8—H8	126.2
C2—Mo1—C8	135.18 (9)	C7—C8—C9	107.6 (2)
C2—Mo1—C9	101.20 (9)	C9—C8—Mo1	72.05 (13)
C3—Mo1—P1	133.99 (6)	C9—C8—H8	126.2
C3—Mo1—C5	117.05 (8)	Mo1—C9—H9	119.2
C3—Mo1—C6	141.34 (8)	C5—C9—Mo1	74.77 (13)
C3—Mo1—C7	117.61 (8)	C5—C9—C8	108.3 (2)
C3—Mo1—C8	85.28 (8)	C5—C9—H9	125.9
C3—Mo1—C9	85.13 (8)	C8—C9—Mo1	72.00 (13)
C5—Mo1—P1	104.19 (6)	C8—C9—H9	125.9
C5—Mo1—C6	34.54 (8)	P1—C10—H10A	108.4
C6—Mo1—P1	84.62 (6)	P1—C10—H10B	108.4
C7—Mo1—P1	100.66 (6)	N1—C10—P1	115.67 (16)
C7—Mo1—C5	58.09 (8)	N1—C10—H10A	108.4
C7—Mo1—C6	34.64 (8)	N1—C10—H10B	108.4
C8—Mo1—P1	136.01 (6)	H10A—C10—H10B	107.4
C8—Mo1—C5	58.94 (9)	P1—C11—H11A	109.8
C8—Mo1—C6	58.33 (8)	P1—C11—H11B	109.8
C8—Mo1—C7	35.35 (8)	N2—C11—P1	109.32 (14)
C8—Mo1—C9	35.95 (9)	N2—C11—H11A	109.8
C9—Mo1—P1	139.10 (6)	N2—C11—H11B	109.8
C9—Mo1—C5	35.20 (9)	H11A—C11—H11B	108.3
C9—Mo1—C6	58.12 (8)	P1—C12—H12A	109.0
C9—Mo1—C7	58.84 (8)	P1—C12—H12B	109.0
C10—P1—Mo1	113.94 (8)	N3—C12—P1	112.74 (15)
C11—P1—Mo1	120.83 (7)	N3—C12—H12A	109.0
C11—P1—C10	98.79 (11)	N3—C12—H12B	109.0
C11—P1—C12	97.73 (10)	H12A—C12—H12B	107.8
C12—P1—Mo1	117.51 (7)	N1—C13—H13A	108.6
C12—P1—C10	105.05 (11)	N1—C13—H13B	108.6
C10—N1—C13	115.65 (18)	N2—C13—N1	114.62 (18)
C15—N1—C10	118.14 (19)	N2—C13—H13A	108.6
C15—N1—C13	126.21 (19)	N2—C13—H13B	108.6
C13—N2—C11	112.11 (17)	H13A—C13—H13B	107.6
C14—N2—C11	112.71 (17)	N2—C14—N3	114.37 (18)
C14—N2—C13	116.44 (18)	N2—C14—H14A	108.7
C12—N3—C14	115.27 (17)	N2—C14—H14B	108.7
C17—N3—C12	118.24 (18)	N3—C14—H14A	108.7
C17—N3—C14	126.23 (19)	N3—C14—H14B	108.7
O1—C1—Mo1	176.4 (2)	H14A—C14—H14B	107.6
O2—C2—Mo1	173.5 (2)	O4—C15—N1	121.4 (2)
O3—C3—Mo1	120.51 (16)	O4—C15—C16	120.1 (2)
O3—C3—C4	117.4 (2)	N1—C15—C16	118.5 (2)
C4—C3—Mo1	122.10 (16)	C15—C16—H16A	109.5
C3—C4—H4A	109.5	C15—C16—H16B	109.5
C3—C4—H4B	109.5	C15—C16—H16C	109.5
C3—C4—H4C	109.5	H16A—C16—H16B	109.5
H4A—C4—H4B	109.5	H16A—C16—H16C	109.5
H4A—C4—H4C	109.5	H16B—C16—H16C	109.5
H4B—C4—H4C	109.5	O5—C17—N3	121.3 (2)
Mo1—C5—H5	122.1	O5—C17—C18	120.1 (2)
C6—C5—Mo1	73.39 (13)	N3—C17—C18	118.6 (2)
C6—C5—H5	126.2	C17—C18—H18A	109.5
C6—C5—C9	107.6 (2)	C17—C18—H18B	109.5
C9—C5—Mo1	70.03 (13)	C17—C18—H18C	109.5
C9—C5—H5	126.2	H18A—C18—H18B	109.5
Mo1—C6—H6	122.2	H18A—C18—H18C	109.5
C5—C6—Mo1	72.07 (13)	H18B—C18—H18C	109.5
			
Mo1—P1—C10—N1	−172.38 (14)	C11—P1—C10—N1	−42.89 (19)
Mo1—P1—C11—N2	176.42 (11)	C11—P1—C12—N3	49.62 (18)
Mo1—P1—C12—N3	−179.56 (13)	C11—N2—C13—N1	67.4 (2)
Mo1—C5—C6—C7	−62.85 (16)	C11—N2—C14—N3	−64.3 (2)
Mo1—C5—C9—C8	64.72 (15)	C12—P1—C10—N1	57.6 (2)
Mo1—C6—C7—C8	−62.18 (15)	C12—P1—C11—N2	−54.99 (16)
Mo1—C7—C8—C9	−64.94 (15)	C12—N3—C14—N2	57.2 (3)
Mo1—C8—C9—C5	−66.55 (15)	C12—N3—C17—O5	2.6 (3)
C5—C6—C7—Mo1	63.00 (15)	C12—N3—C17—C18	−177.9 (2)
C5—C6—C7—C8	0.8 (2)	C13—N1—C10—P1	46.5 (2)
C6—C5—C9—Mo1	−64.27 (15)	C13—N1—C15—O4	−173.8 (2)
C6—C5—C9—C8	0.5 (2)	C13—N1—C15—C16	8.8 (3)
C6—C7—C8—Mo1	64.41 (15)	C13—N2—C11—P1	−67.49 (19)
C6—C7—C8—C9	−0.5 (2)	C13—N2—C14—N3	67.3 (2)
C7—C8—C9—Mo1	66.60 (15)	C14—N2—C11—P1	66.2 (2)
C7—C8—C9—C5	0.0 (2)	C14—N2—C13—N1	−64.5 (2)
C9—C5—C6—Mo1	62.07 (15)	C14—N3—C12—P1	−53.5 (2)
C9—C5—C6—C7	−0.8 (2)	C14—N3—C17—O5	−171.1 (2)
C10—P1—C11—N2	51.64 (16)	C14—N3—C17—C18	8.4 (3)
C10—P1—C12—N3	−51.70 (19)	C15—N1—C10—P1	−133.89 (18)
C10—N1—C13—N2	−55.1 (3)	C15—N1—C13—N2	125.4 (2)
C10—N1—C15—O4	6.6 (3)	C17—N3—C12—P1	132.06 (18)
C10—N1—C15—C16	−170.7 (2)	C17—N3—C14—N2	−128.9 (2)

### trans-Acetyldicarbonyl(η5-cyclopentadienyl)(3,7-diacetyl-1,3,7-triaza-5-phosphabicyclo[3.3.1]nonane)molybdenum(II) (2) Hydrogen-bond geometry (Å, º)

**Table:**

*D*—H···*A*	*D*—H	H···*A*	*D*···*A*	*D*—H···*A*
C7—H7···O4^i^	0.93	2.45	3.353 (3)	163
C11—H11*A*···O5^ii^	0.97	2.41	3.211 (3)	140
C12—H12*B*···O4^ii^	0.97	2.60	3.409 (3)	141
C13—H13*A*···O5^iii^	0.97	2.57	3.474 (3)	156
C13—H13*B*···O3^iv^	0.97	2.46	3.261 (3)	139

Symmetry codes: (i) −*x*+1, −*y*+1, −*z*+1; (ii) −*x*+1/2, *y*−1/2, −*z*+1/2; (iii) *x*+1/2, −*y*+1/2, *z*+1/2; (iv) *x*−1/2, −*y*+1/2, *z*+1/2.
